# Impact of gas humidification and nebulizer position under invasive ventilation: preclinical comparative study of regional aerosol deposition

**DOI:** 10.1038/s41598-023-38281-9

**Published:** 2023-07-08

**Authors:** Yoann Montigaud, Quentin Georges, Lara Leclerc, Anthony Clotagatide, Aurore Louf-Durier, Jérémie Pourchez, Nathalie Prévôt, Sophie Périnel-Ragey

**Affiliations:** 1 Mines Saint-Etienne, Univ Jean Monnet, INSERM, U1059 Sainbiose; Centre CIS, 42023 Saint-Etienne, France; 2 Intensive Care Unit G, CHU Saint-Etienne, 42055 Saint-Etienne, France; 3 Nuclear Medicine Unit, CHU Saint-Etienne, 42055 Saint-Etienne, France; 4 Université Jean Monnet, Mines Saint-Etienne, INSERM, U1059 Sainbiose, 42023 Saint-Etienne, France; 5 Intensive Care Unit G, Saint Etienne University Hospital, North Hospital, UMR INSERM U1059, Avenue Albert Raymond, 42270 Saint Priest en Jarez, France

**Keywords:** Drug delivery, Biomedical engineering, Asthma, Chronic obstructive pulmonary disease, Cystic fibrosis, Respiratory distress syndrome

## Abstract

Successful aerosol therapy in mechanically ventilated patients depends on multiple factors. Among these, position of nebulizer in ventilator circuit and humidification of inhaled gases can strongly influence the amount of drug deposited in airways. Indeed, the main objective was to preclinically evaluate impact of gas humidification and nebulizer position during invasive mechanical ventilation on whole lung and regional aerosol deposition and losses. Ex vivo porcine respiratory tracts were ventilated in controlled volumetric mode. Two conditions of relative humidity and temperature of inhaled gases were investigated. For each condition, four different positions of vibrating mesh nebulizer were studied: (i) next to the ventilator, (ii) right before humidifier, (iii) 15 cm to the Y-piece adapter and (iv) right after the Y-piece. Aerosol size distribution were calculated using cascade impactor. Nebulized dose, lung regional deposition and losses were assessed by scintigraphy using ^99m^technetium-labeled diethylene-triamine-penta-acetic acid. Mean nebulized dose was 95% ± 6%. For dry conditions, the mean respiratory tract deposited fractions reached 18% (± 4%) next to ventilator and 53% (± 4%) for proximal position. For humidified conditions, it reached 25% (± 3%) prior humidifier, 57% (± 8%) before Y-piece and 43% (± 11%) after this latter. Optimal nebulizer position is proximal before the Y-piece adapter showing a more than two-fold higher lung dose than positions next to the ventilator. Dry conditions are more likely to cause peripheral deposition of aerosols in the lungs. But gas humidification appears hard to interrupt efficiently and safely in clinical use. Considering the impact of optimized positioning, this study argues to maintain humidification.

## Introduction

Since more than 30 years, intensive care allowed to increase survival rate of patients in critical state. Despite this tremendous success, some of clinical practices in intensive care units (ICU) are still led by empirical choices instead of evidence-based decisions. Among these, nebulization practices in ICU rest on main data from old in vitro studies^1^ while it could be depicted as essential interest considering that 25% of ICU patients receive nebulization of various drugs^2,3^.

Different parameters affect lung deposition of therapeutic aerosol during invasive mechanical ventilation. First, it can be related to the patient (disease, pulmonary mechanics etc.), but also to the couple drug-nebulizer (physicochemical properties of drug, performances of nebulizer etc.), and lastly to the ventilator and its circuit (tuning and triggering, heat and humidification of inhaled gas, position of nebulizer on inspiratory limb etc.)^4–6^. Influence of these parameters, in term of aerosol delivery to the patient, are mainly assessed by in vitro studies widely performed by measure of the aerosol impaction on a filter at the end of the ventilator circuit. This point could lack of relevance because it differs from administration in the desired location in the respiratory tract (RT). Moreover, expiratory loss, cannot be assessed due to the use of filters to collect aerosol^7,8^. Besides, clinical studies are mainly focused on patients’ outcomes, such as the therapeutic efficacy of treatment^7,9–13^. Indeed, literature is scarce concerning the impact of humidification and nebulizer position on the regional aerosol deposition in patients under mechanical ventilation^14,15^. Moreover, these rare published data lack of systematic assessment and should be cautiously extrapolated due to the wide range of clinical practices, for both ventilator tuning, nebulization devices used and aerosolized drug delivery^2,15–17^.

Therefore, a data gap needs to be bridged regarding the specific knowledge devoted to regional aerosol deposition in mechanically ventilated patients^1^. However, technical constraints and ethical restrictions limit clinical research on ICU patients. Moreover, existing in vivo preclinical models sometimes lack of relevance (e.g. ventilation physiology of rodents is very different from humans^18–22^) or are difficult to compare with in vivo human data.

To overcome these limitations, we previously developed and validated an ex vivo preclinical model of porcine RT for aerosol deposition studies in spontaneous^23,24^ and mechanical ventilation conditions^25^. Considering reproducibility, ethical, anatomical and physiological interests of this preclinical model, useful data can be produced to implement aerosol therapy practices in ICUs. Consequently, the present study aims to assess the impact of gas humidification and of the nebulizer position in the ventilator circuit on the regional aerosol deposition as well as in the different elements composing the ventilation set up with this ex vivo model.

## Methods

### Study design

**Figure 1:**
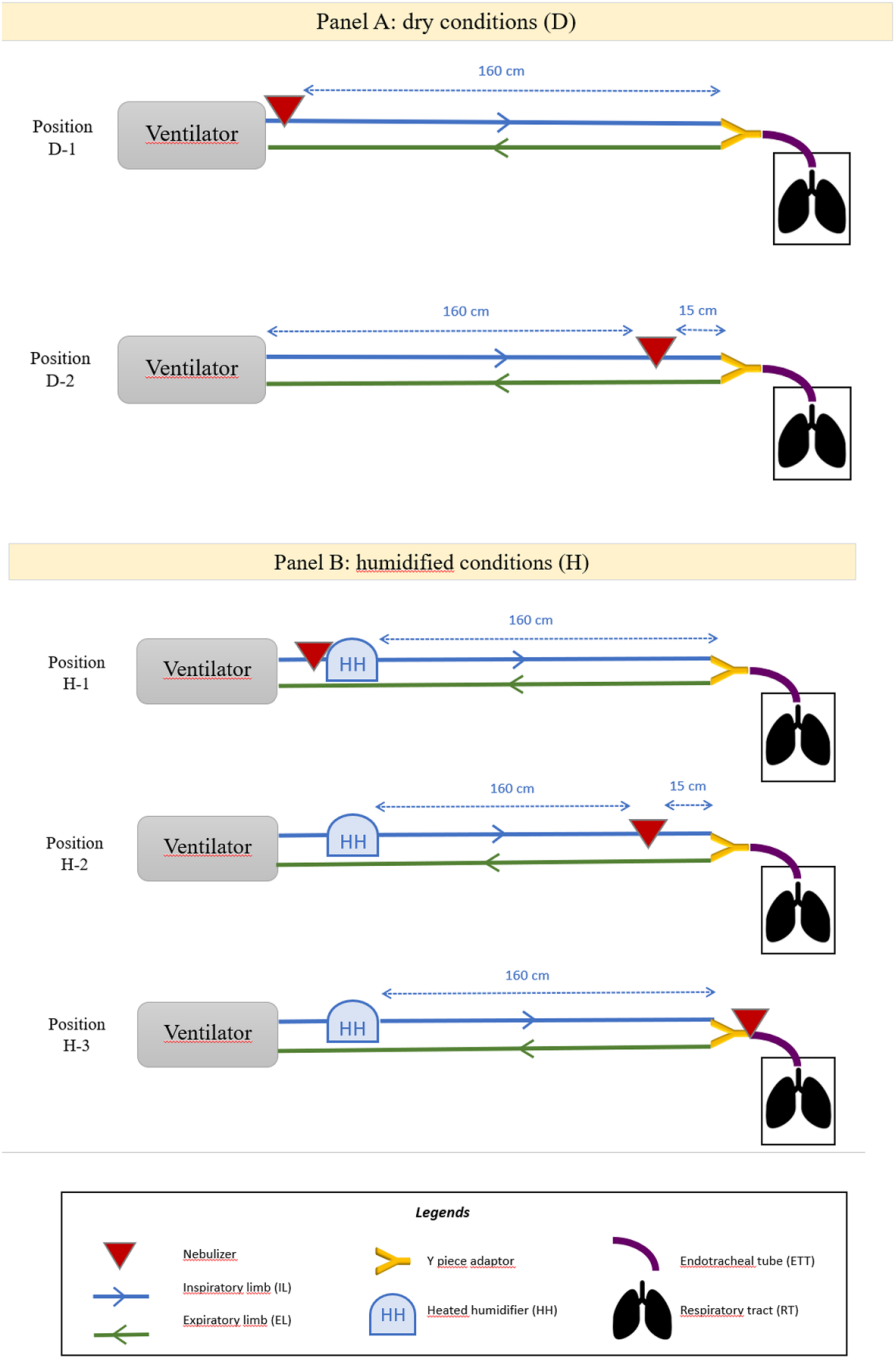
Schematic representation of the different experimental conditions according to humidification and nebulizer position. Panel (**A**) dry conditions (D); Panel (**B**) humidified conditions (H). *Cm* centimeters.

This aerosol deposition study is based on an ex vivo RT model previously validated for mechanical ventilation applications^25^ and allowing a scintigraphic regional aerosol deposition study of each part of the ventilation set up. Reproducibility has been checked and consequently each experimental condition was tested at least in 3 replicates with different porcine RT. This study is composed of two categories of experiments: part D corresponds to dry and cold conditions i.e. no humidification nor heating of gases in the circuit. Part H corresponds to humidified and heated conditions. This latter was achieved thanks to a heated humidifier (HH). Figure 1 displays schematically the different experimental conditions. According to different recommendations on nebulization and to manufacturer, 3 positions were chosen: first next to ventilator or previous HH. The second position was 15 cm prior the Y piece adaptor and the third just after this latter, at the end of endotracheal tube (ETT). Consequently, 5 different experimental conditions are compared in this work. The main endpoint is the scintigraphic quantification of aerosol deposition in each component.

### Preclinical respiratory model

Aerosol delivery during invasive mechanical ventilation using a preclinical ex vivo respiratory model was previously fully validated^25^. It was composed of a porcine RT collected from slaughterhouses as waste of food industry. Hence, they succeed all the sanitary controls in accordance with French regulation with strict veterinarian controls of animal welfare and any animal was specifically sacrificed for this work, in accordance with the 3R guidelines^26^. For sanitary reasons, each respiratory tract was used within 24 h. Otherwise, they were frozen and thereafter thawed at ambient temperature few hours before the experiment. Relevant sutures were realized on each significant cut and their number was recorded. A systematic bronchoscopy allowed to ensure the absence of major obstruction in reachable bronchi. Respiratory tracts were placed in a sealed enclosure. The latter allows both positive and negative pressure ventilations. A 7.5 mm inner diameter ETT (Smiths Medical, Portex^®^ SACETT™ Plymouth Minnesota, United States) with a 34 cm length was connected to the trachea with a standardized trachea length of 15 cm to avoid selective intubation. The model was set in a 30° proclive position, as close as possible to the patient position for nebulization in ICU and mechanically ventilated with positive airway pressure.

### Ex vivo model and ventilation settings

The ex vivo model was used under controlled volumetric ventilation without triggering, thanks to a medical ventilator [ICU Evita 4 ventilator (Dräger, Germany)]. Systematic security tests were performed before experiments. An important data is the absence of bias flow for this ventilator according to manufacturer. This ventilation mode was selected because it is currently recommended to optimize RT deposition fraction of nebulization^1^ and is widely used in ICUs^27^. The ventilation parameters were identical for each condition and adapted to be comparable to Ehrmann et al*.*^28^ with a tidal volume of 540 mL, respiratory rate of 20/min, inspiratory flow of 35 L/min with a constant flow pattern, inspiratory to expiratory ratio of 1/2, positive end expiratory pressure of 9 cmH_2_O and inspired oxygen fraction of 21%. These parameters were chosen in accordance with data from a large cohort study in ICUs over the world^27^. For this work, no spontaneous breathing was used. The ETT was set with an angulation of 90°, determined thanks to data from CT scans of intubated patients.

The ventilator was connected to the endotracheal tube with a medical 2 limbs circuit (Evaqua 2™, Fischer&Paykel Healthcare, Auckland, New Zealand) with or without heated humidifier (HH, MR410, Fischer&Paykel Healthcare, Auckland, New Zealand) in the inspiratory limb according to each experimental condition studied. The first part of this study corresponds to dry and cold conditions (conditions D) i.e. without HH. These were tested each time with a different circuit, without any humidifier in line. The second part corresponds to humidified and heated conditions (conditions H) thanks to the HH placed on the circuit as recommended by manufacturer. The humidifier was turned on at least 15 min before nebulization. An expiratory filter (DAR™ Heat and Moisture Exchangers (HME) Filter, ref. 354/5876, Covidien™, Dublin, Ireland) was systematically placed on the expiratory limb, close to the expiratory valve to avoid damages and obstructions, as recommended for nebulization^28^.

### Nebulization procedure

A vibrating mesh nebulizer was used (Aerogen Solo^®^, Aerogen Limited, Galway, Ireland) because among all nebulization devices; vibrating mesh are those leading to the best percentage of nominal dose nebulized^29,30^. The minimal duration of nebulization was standardized to 15 min based on preliminary test run, in good accordance with previous study from Dugernier et al.^15^ showing an average 9 min (± 3 min) nebulization. The ending of nebulization was checked systematically before acquisitions. According to each experimental condition, the nebulizer was placed at four different positions on the respiratory circuit: next to the ventilator, right before HH, 15 cm from Y-piece adapter and right after the latter (Fig. 1). Each experimental condition was tested at least in 3 replicates. The condition H-1 corresponds to experiments previously published^25^, and was performed with 6 replicates to check reproducibility of the experiment.

### Scintigraphic assessment of regional aerosol deposition

The nebulizer was filled with 3 mL of ^99m^technetium-labeled diethylene-triamine-penta-acetic acid (^99m^Tc-DTPA, 100 MBq commercial kit Technescan DTPA, Curium, Netherlands) for acquisition of planar scintigraphic images. The latter (matrix 256 × 256) were recorded with a variable angle dual detector Single Photon Emission Computed Tomography with a Computed Tomography scan (SPECT/CT—SYMBIA T2; Siemens, Knoxville, United States) equipped with a low-energy, high-resolution collimator (FWHM 8.3 mm at 10 cm); tested weekly for uniformity (UFOV 533 mm × 387 mm, CFOV 400 mm × 290 mm). First, the initial radioactive dose filled in the nebulizer was quantified (scintigraphic images, 180-s anterior/posterior, were acquired corresponding to the fully loaded minus the empty syringe). Nominal dose stood for the total amount of radioactivity introduced into the nebulizer, allowing to determine the nebulized fraction of nominal dose, i.e. the output of the nebulizer. Then, the nebulization procedure was performed. Finally, 180-s anterior/posterior images of each part of the experimental setup were acquired for each element: empty nebulizer, inspiratory limb, heated humidifier, endotracheal tube, Y-piece associated to expiratory limb and RT. Regarding the latter element, two different regions of interest (ROIs) were identified on scintigraphies within each lung to divide lungs into central and peripheral zones. A nuclear medicine physician used a semi-automatic delineation method with a variable threshold based on adaptative signal to background ratio and manual outlines the central region (trachea ad stem bronchi). This allowed to calculate the relative uptake of central and peripheral regions for both left and right lung. ROIs were delimited on the images with a correction considering the background radiation, the physical decay of radioactivity and tissue attenuation correction factors. Consequently, as stated by Newman et al.^31–33^, some limitations of data quantification in planar imaging are inherent to its two-dimensional nature. It is accepted that errors of no more than 10% in the quantification of whole-lung deposition are tolerated and that mass balance close to 100% validate the method used for attenuation and scatter correction. Hence, the results were expressed as a percentage of activity in relation to the nebulized dose, adjusted to 100%.

### Aerosol size distribution

To determine the aerodynamic size distribution of the aerosol, the European Pharmacopoeia indicates to use the ACI (Andersen Cascade Impactor as described in the European Pharmacopeia—Monograph 2.9.18^34^). The ACI holds 8 stages from 0.4 to > 9 µm and is operated at 28.3 L/min with a vacuum pump (Low Capacity Pump Model LCP5, Copley Scientific Limited, Nottingham, United Kingdom). 3 mL of Sodium Fluoride solution at 2.5% (m/V) were introduced into the tank of the nebulizer. The nebulization process continues until the volume left in the nebulizer is over, which basically leads the nebulizer to cease functioning continuously and begins to sputter. For D and H experimental conditions, the ACI was placed after the Y piece, right before ETT. After nebulization, deposited fractions on each stage of the ACI were collected and re-suspended in 5 mL of deionized water, then 250 µL of TISAB IV solution (Total ionic strength adjustment buffers, Sigma-Aldrich, Saint-Louis, United States) were added. Concentrations of fluoride ions were determined with a ionometer (SevenGo Pro apparutus and Perfection probe, Mettler Toledo, Columbus, United States). Calibration of the apparatus was performed before each experiment. Mathematical determination of MMAD (Mass Median Aerodynamic Diameter) was performed with Excel software (Microsoft Excel, Microsoft, Redmond, United States).

### Statistical analysis

Results are expressed as mean percentage (%) ± standard deviation (SD) of the nebulized dose of radioactivity. Statistical analyses were performed using GraphPad Prism^®^ v8.0.2 (San Diego, United States).

Comparison of respiratory tracts used, in terms of mass and cuts, for each position was performed with a Kruskall–Wallis’ test and post-hoc Dunn’s multiple comparison test.

To compare the deposited fractions on each part of the model, a 2-way analysis of variance (ANOVA) was carried out with a post-hoc Tukey’s multiple comparison test. Comparison of central and peripheral deposited fractions were carried out with a 2-way ANOVA with post-hoc Tukey’s multiple comparison tests. Comparison of MMAD were performed with 1-way ANOVA with post-hoc Tukey’s multiple comparison test.

### Ethical approval

Ethical approval is not applicable according to French regulation as RT were collected from slaughterhouses as waste of food industry. Hence, any animal was specifically sacrificed for this work and they succeed all the sanitary controls in accordance with French regulation with strict veterinarian controls of animal welfare.

## Results

### Nebulization

The model was set up 18 times with different RT achieving successful ventilation and nebulization. 6 experiments were realized for Position D-1, and 3 experiments for conditions (i.e. D-2, H-1, H-2 and H-3). Nebulization ended successfully before acquisitions for all experiments.

The nebulized fraction of nominal dose was 94 ± 7% for position D-1, 98 ± 1% for position D-2, 91 ± 12% for position H-1, 97 ± 3% for position H-2, 96 ± 4% for position H-3. Comparisons showed no significant difference in nebulizer output (i.e., in amount of radiotracer nebulized) according to the condition. Nebulized dose for experimental condition are displayed in Table 1*.*

**Table 1:** Nebulized fractions of nominal dose for each condition. Nominal dose is the dose loaded in the nebulizer. Comparisons showed no significant difference.

Condition	Nebulized dose
D-1	94% (± 7%)
D-2	98% (± 2%)
H-1	91% (± 12%)
H-2	97% (± 3%)
H-3	96% (± 4%)

### Deposited fractions

**Figure 2:**
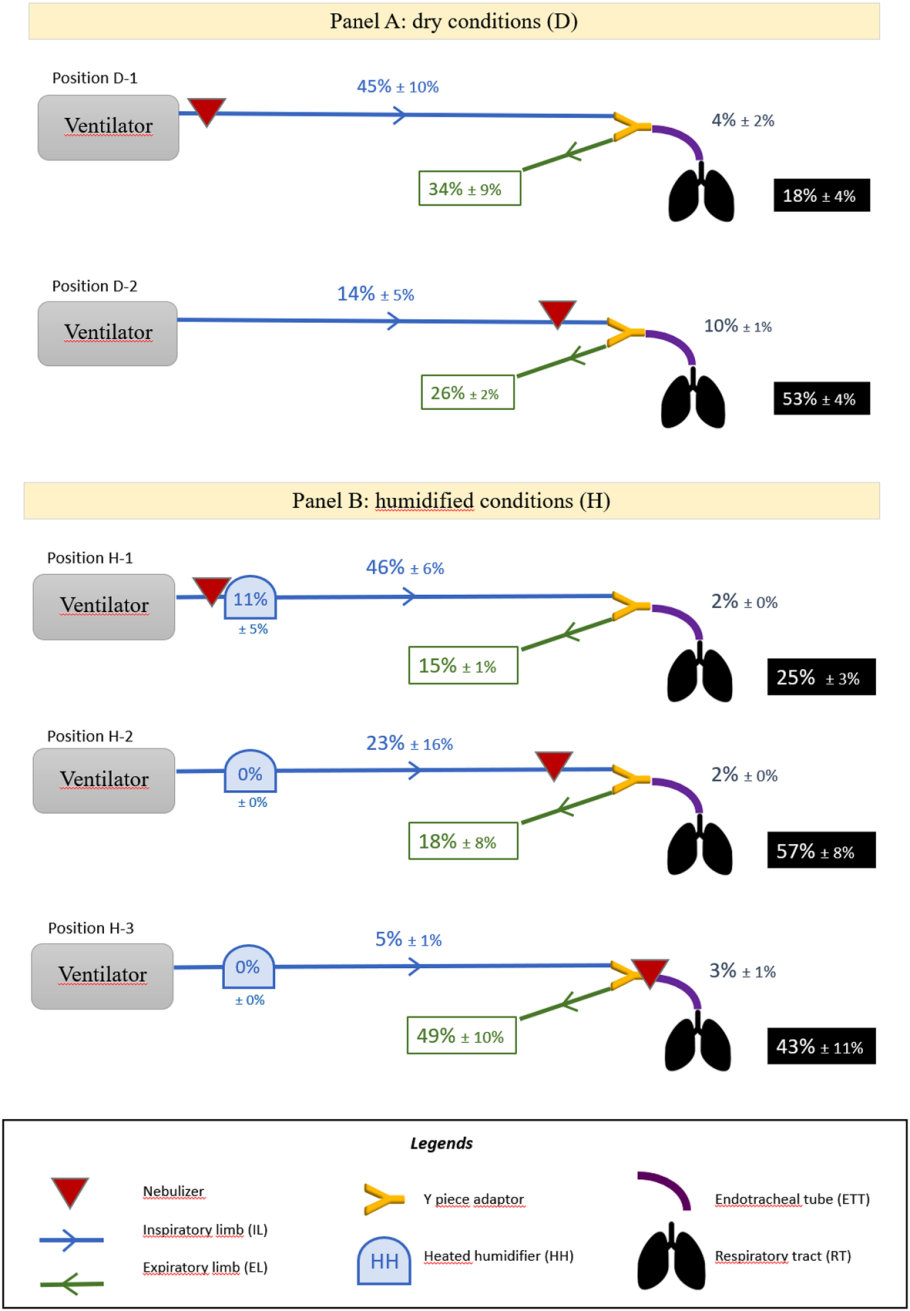
Deposited fractions (%) of the nebulized dose in each component of the setting. Panel (**A**) dry and cold conditions; Panel (**B**) heated and humidified conditions. Results expressed as mean ± standard deviation.

The scintigraphic images performed allowed to calculate the deposited fractions for each component of the setting, leading to comparative results as shown in Fig. 2 and in supplementary data Fig. S1. For D-1 and D-2 experiments in dry and cold conditions, deposited fractions significantly varied according to the nebulizer positions. They reached for D-1 (respectively for D-2) 45 ± 10% (respectively 14 ± 5%) for inspiratory limb, 4 ± 2% (respectively 10 ± 1%) for endotracheal tube, 18 ± 4%) (respectively 53 ± 4%) for the RT, and finally 34 ± 9%) (respectively 26 ± 2%) for expiratory limb.

For heated and humidified conditions, i.e. positions H-1, H-2 and H-3, deposited fractions were respectively 46% (± 6%), 23% (± 16%) and 5% (± 1%) for inspiratory limb, 2% (± 0%), 2% (± 0%) and 3% (± 1%) for endotracheal tube. For the RT they reached respectively 25% (± 3%), 57% (± 8%) and 43% (± 11%). Finally, for expiratory limb values were respectively 15% (± 1%), 18% (± 8%) and 49% (± 10%).

For H-1, H-2 and H-3 experiments in the heated and humidified condition, deposited fractions were respectively 46 ± 6%, 23 ± 16% and 5 ± 1% for inspiratory limb, 2 ± 0%, 2 ± 0% and 3 ± 1% for endotracheal tube. For the RT they reached respectively 25 ± 3%, 57 ± 8% and 43 ± 11%. Finally, for expiratory limb values were respectively 15 ± 1%, 18 ± 8% and 49 ± 10%.

As presented in Table 2 and on Fig. 2A, RT fraction of nebulized dose varied significantly according to the experimental condition. No significant difference arose for RT deposition fractions between the two conditions next to Y-piece adapter (Conditions H-2 and H-3) and between dry and humidified conditions for a same position. By contrast, a great impact of the nebulizer position was noticed. Indeed, we showed significant differences for quantitative RT deposition with significantly around two times lower RT deposited fraction at proximal nebulizer position on inspiratory limb (conditions D-1 and H-1) comparatively at distal nebulizer positions on inspiratory limb (conditions D-2, H-2, and H-3). All p values for these comparisons are available as Supplementary Table S1.

**Table 2:** Fraction of the nebulized dose deposited in the respiratory tract (RT) and its repartition. Conditions D: dry conditions; Conditions H: humidified conditions. Results are displayed as mean and standard deviation (SD). All data are expressed as percentages of nebulized dose.

Condition	RT	Central	Peripheral
D-1	18% (± 4%)	3% (± 2%)	15% (± 4%)
D-2	53% (± 4%)	29% (± 2%)	24% (± 5%)
H-1	25% (± 3%)	13% (± 0%)	11% (± 3%)
H-2	57% (± 8%)	39% (± 7%)	18% (± 3%)
H-3	43% (± 11%)	26% (± 6%)	17% (± 5%)

### Regional aerosol deposition

**Figure 3:**
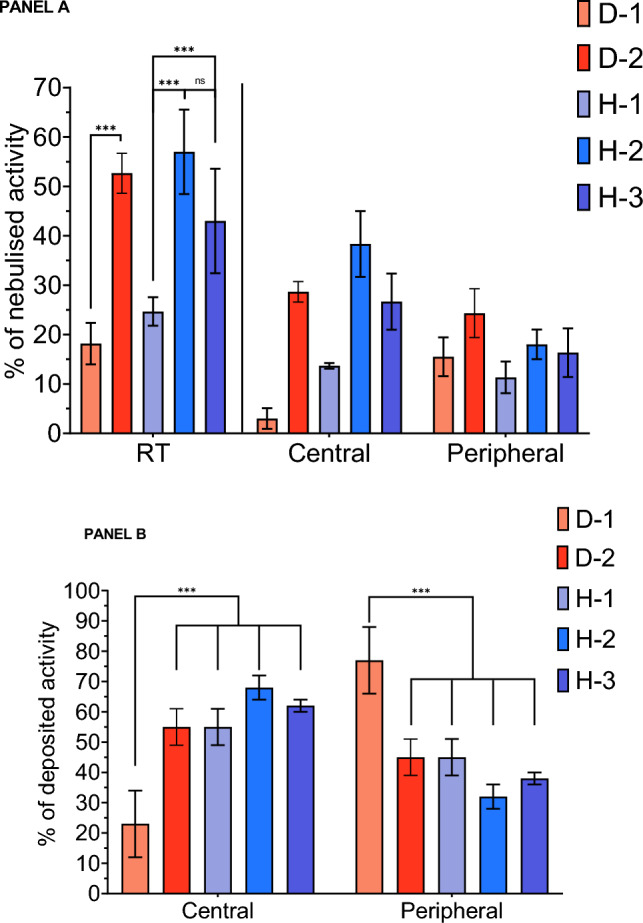
Bar graph of deposited activity in respiratory tract (RT), central and peripheral regions of interest. Panel (**A**) comparison for each condition of RT, central and peripheral fractions of nebulized dose. Panel (**B**) comparison of central and peripheral activities expressed as percentages of RT deposited activity. Conditions D in red colors: dry conditions; Conditions H with blue colors: humidified conditions. Results expressed as mean ± standard deviation. *p < 0.05; **p < 0.01; ***p < 0.001. *ns* non-significant.

Results of regional aerosol distribution are displayed in Table 2 and Fig. 3A, expressed as fractions of nebulized dose. As previously described the RT deposited dose vary widely according to the condition. Consequently, it is of importance to consider the regional aerosol deposition as percentages of RT deposited activity to compare results. These results are resented for each condition on Fig. 3B. Briefly, for dry conditions D-1 and D-2, the central part of RT deposited fractions were respectively 16 ± 11% and 55 ± 6%. For humidified conditions H-1, H-2 and H-3, the central part of RT fractions were respectively 55 ± 6%, 68 ± 4% and 62 ± 2%. The comparisons performed showed, again, no significant difference between dry and humidified conditions. However, the interaction of nebulizer position and gas humidification lead to a significant difference in RT distribution for condition D-1 compared to the other experimental conditions (p < 0.001). In fact, the regional distribution of aerosol for condition D-1 was significantly more peripheral than all other. All p values for these data are available as Supplementary Table S2.

### Aerosol size distribution

MMAD for each experimental condition are displayed in Table 3*.* The MMAD measured for the nebulizer used in this work was 1.94 ± 0.34 µm. For dry conditions D-1 and D-2 the MMAD reached respectively 1.69 ± 0.00 µm and 1.92 ± 0.09 µm. For humidified conditions H-1, H-2 and H-3 the calculated MMAD were significantly higher with respectively 4.12 ± 0.04 µm, 4.79 ± 0.34 µm and 4.94 ± 0.27 µm.

**Table 3:** Mass median aerodynamic diameter (MMAD) of the nebulizer and for each experimental condition. Conditions D: dry conditions; Conditions H: humidified conditions. Results are displayed as mean and standard deviation (SD).

Condition	MMAD ± SD
Nebulizer	1.94 ± 0.34 µm
D-1	1.69 ± 0.00 µm
D-2	1.92 ± 0.09 µm
H-1	4.12 ± 0.04 µm
H-2	4.79 ± 0.34 µm
H-3	4.94 ± 0.27 µm

The comparison of MMAD for the nebulizer alone and both dry conditions (D-1 and D-2) showed no significant statistical differences. In contrast, each humidified condition had a MMAD significantly greater from nebulizer alone and dry conditions. Moreover, the condition H-1 showed a MMAD significantly lower from H-2 and H-3, although no significant statistical difference was observed between conditions H-2 and H-3. The Supplementary Table S3 presents all the p value for these results.

## Discussion

To the best of our knowledge, this is the first study comparing the impact of nebulizer position and gas humidification on the regional aerosol deposition using an innovative and reproducible preclinical respiratory model. This work showed with comparative conditions that these two experimental parameters can greatly change the mean deposited fractions in the RT from 18 to 55%, leading to potential wide differences in drug delivery to mechanically ventilated patients. This finding raises a major concern about practices in ICU as some international epidemiological studies highlighted disparate clinical practices^2^. Moreover, this study was performed with a new generation vibrating mesh nebulizer showing a mean aerosol output higher than 90%. This point agrees with previous data^35^. This is of high relevance because epidemiological studies show a majority use in ICU of less efficient jet nebulizers^2,36^. For example Ehrmann et al. observed 10% of vibrating mesh nebulizer and 56% of jet nebulizers use in an international study^2^. Despite economic considerations, their output is largely lower, ranging from 30 to 55% according to different studies^35,37^. Consequently, considering wide differences in aerosol output depending on nebulizer technologies and the great impact of the aerosol device position on the ventilator circuit and gas humidification, the potential cumulative differences could lead to clinical treatment failure independently of drug efficiency. Finally, the type of nebulizer has been suspected to impact ICU patients outcome as a trends to shorter length of stay for asthmatic patients^10^.

### Impact of nebulizer position in the ventilator circuit

The location of the nebulizer in ventilator circuit is a major determinant for the drug dose reaching the RT^38^. Indeed, despite a manufacturer recommendation^39^, position upstream the heated humidifier (HH), (conditions D-1 and H-1) led in this study, to a mean RT deposited fraction ranging from 18 to 25% while a position right before the Y-piece adapter (conditions D-2 and H-2) produced a mean RT deposited fraction ranging from 53 to 57%. Obviously, this difference is largely significant and can lead to wide differences in the treatment efficacy. However, Moraine et al. failed to find a clinical difference while measuring ipratropium in urine after nebulization in mechanically ventilated patients^40^. According to our results, evaluating separately the impact of gas humidification and of nebulizer position on a standardized preclinical respiratory model, the lake of significant difference for Moraine et al. could be due to the interaction of both factors. Indeed, they compared whether nebulizer prior the HH or nebulizer right before the Y-piece adapter in dry conditions, leading to potential interaction of these factors. Even if the difference is not significant, the nebulizer position beyond the Y-piece adapter tend to a higher loss in expiratory limb and lower RT deposited fraction than the position 15 cm before Y-piece adapter (p = 0.0508). These data are in accordance with the in vitro study from Ari et al.^7^ which compared the three same positions with and without gas humidification on an in vitro model. Nevertheless, due to a design with deposition on a filter instead of a RT, the expiratory loss and regional deposition could not be evaluated on their model. The expiratory loss is described as the major drawback for positions closed to expiratory limb^38^ this is consequently an important interrogation. On the other hand, position 2 is characterized by the 15 cm before the Y-piece, serving as a reservoir for aerosol continuously generated during expiration. This position is often recommended for this effect^4,12,30,41–46^. Finally, this study finds an optimal position at 15 cm before the Y-piece adapter for the vibrating mesh nebulizer independently from humidification, and this result is confirmed even considering the expiratory loss. As this is different from manufacturer recommendations^39^ for this specific nebulizer, the intensivists have to be aware of this point.

### Impact of gas humidification

No difference was shown for humidification in terms of absolute RT deposited dose. This point differs from previous data on the humidification impact on aerosol delivery, as an important decrease, around 40%, is widely described^47^. This could be due to a difference between nebulizer types, as vibrating mesh nebulizers are relatively new technologies. Moreover, Ari et al., using the same nebulizer than us, found a significant decrease with humidification for the positions before the Y-piece adapter but no difference after this latter^7^. More recently, Ashraf et al., comparing conditions very similar to D-1 and H-1 in our study, i.e. the same vibrating mesh nebulizer close to the ventilator with or without humidification, found very similar results with inhalable doses around 20% equally^35^. Nevertheless, a difference arose in our results for RT distribution when the position upstream HH and dry condition interacts. Indeed, these parameters (condition D-1) allowed a more peripheral deposition compared to each other condition, as previously described^25^. However, this finding must be first balanced with an absolute RT deposited fraction lower than other conditions and then with the risks for patients when interrupting the humidification for nebulization. Indeed, there are two principal ways of humidification for patients under mechanical ventilation: heat and moisture exchangers needing to be removed for the duration of nebulization to allow the passing of aerosol^48^ and HH. The cessation of HH just before nebulization showed an important residual humidity in limbs, being consequently ineffective to obtain “dry conditions”^29^ and the removal of humidification was reported as dangerous for patients if prolonged^49,50^. The humidification question has consequently to be seen as security factor for patients mechanically ventilated. Our finding is in accordance with a clinical study on asthmatic patients showing no significant difference for clinical endpoints according to humidification or not^10^. This point raises questions about evidence to interrupt humidification during nebulization with this vibrating mesh nebulizer.

### Aerosol size distribution

Interestingly, the findings in this study differ in terms of aerosol size distribution from Ashraf et al. data^35^. Indeed, they found no significant difference in MMAD between dry and humid conditions with the same type of vibrating mesh nebulizer (MMAD ranging from 1.90 ± 0.14 µm for humid condition to 1.57 ± 0.05 µm). These discrepancies, while puzzling at first, could be explained by the sampling choices made that differ from our own sampling method. Indeed, as Ashraf et al*.* chose to sample at the distal extremity of the endotracheal tube, we decided to sample the aerosol at the extremity of the Y-piece. However, this is not sufficient to explain these discrepancies. Another point that differs between the studies is the vacuum parameters applied to sample the aerosol. To determine the size distribution, Ashraf et al. decided to apply physiological-like sampling of the aerosol with a vacuum parameter similar to standard breathing pattern found in humans. In this study, we decided to follow our bench protocol for aerosol size distribution according to European Pharmacopeia monography, with flows less based on physiology than in optimized conditions for physical bench comparisons. Therefore, these discrepancies are explained by the fact that we did not measure the same aerosol distribution. On one hand, Ashraf et al. presented granulometry data more focused on the granulometry of droplets that would efficiently reach out the distal extremities of the endotracheal tube. This method is likely to exclude the droplets which would be sedimented during the expiratory phase of the breathing-like sampling pattern, which would decrease aerosol size distribution as the biggest droplets would be excluded from the analysis. On the other hand, the data presented in this study are more focused on assessment of aerosol size distribution on bench with standardized parameters allowing the robust comparison of different nebulizers without exclusion of the biggest droplets composing the aerosol. One could argue on the advantages of one method over the other. Such assertion should be made with caution as the two sampling methods differ on the focus of the analysis. Ashraf et al. method is more patient-based, as measuring fraction reaching the respiratory tract while the method presented in the study is more device-based, not considering deposition in the ETT.

### Strength and limitations

Despite its interesting results, this work presents some limitations. First, as all studies using models, our ex vivo RT will never be able to mimic the complexity of a whole organism. Therefore, as preclinical data, they need to be confirmed by clinical studies. To date, results from this study seem in good agreement with the clinical study on ICU patients of Klockare et al.^14^ where with an ultrasound nebulizer (MMAD 4.0 µm) and gas humidification, the central fraction of RT deposited fraction had a median of 62.8%. Moreover, considering that a vast majority of data for aerosol therapy provide from in vitro studies, this work adds important new information. Indeed, by the adjunction of a RT with an anatomy very close to human’s, data on regional deposition in the RT and exhaled particles are considered. Second, the model used is based on porcine RT collected from slaughterhouses and any animal was specifically sacrificed for this study. This point is equally a limitation because they presented cuts that generated leakage. This particular point was discussed on the previously published data and did not influence the homogeneity of the ventilation^25^. Moreover, improvements were realized to minimize the potential leakage using cyanoacrylate glue and stitches and by a better sealing of the enclosure.

As previously stated, there is a lack of published clinical literature concerning the impact of the inhaled gas humidification and the nebulizer position on the deposition pattern of an aerosol when administered during mechanical ventilation. Among the few clinical studies, the endpoints mainly concern patients’ outcomes, such as improvement of the general state, weaning of mechanical ventilation, etc. or have difficulties to show differences, probably because of the multiplicity of influencing factors^40,51^. Moreover, most of the studies were conducted on patients under non-invasive ventilation, which is a different condition. Hence, more than 30 years after the development of ICU and aerosoltherapy, the nebulizer position in the ventilation circuit is mainly driven by empirical practices, as well as the practices concerning the humidification during nebulization. Moreover, nebulizer technologies have changed as well as ventilators and ventilation parameters. Therefore, there is a need for preclinical studies with a good reproducibility as observed here, allowing to compare humidification and position as isolated factors but equally with their interaction. Finally, these comparisons are quantitative but equally qualitative on the repartition in the RT. To our knowledge, this is the first study allowing all these comparisons.

## Conclusions

Inhaled gas humidification and nebulizer position in the ventilator circuit are two major determinants to optimize aerosol deposition by nebulization under mechanical ventilation. The position 15 cm before the Y-piece adapter allows a more than two-fold higher dose reaching the RT. This result, even needing additional clinical data, is of importance to standardize practices for aerosol delivery in ICU with optimal conditions.

Gas humidification tends to a more central aerosol deposition in the RT but as the interruption can be dangerous for patients and considering the low impact compared to the nebulizer positioning, the removal of HH has to be examined carefully before considering its recommendation. Moreover, considering the impact of nebulizer technology and this optimized position in the ventilator circuit, this study leads to think about pursuit of humidification as a security recommendation.

To conclude, these data are preclinical and consequently need clinical confirmation. Yet, the standardized ex vivo respiratory model used with comparative data of each parameter i.e. humidification and nebulizer position, leads to consider these results for further works on aerosol therapy in ICU and need to be acknowledged by ICU clinicians.

## Supplementary Information

Supplementary Information.

## Data Availability

All main data are available in the main text or the supplementary materials. Complementary information upon data used in the analysis are available on reasonable request to corresponding author.

## Abbreviations

ACI: Andersen cascade impactor
ANOVA: Analysis of variance
DTPA: Diethylene-triamine-penta-acetic acid
ETT: Endotracheal tube
HH: Heated humidifier
HME: Heat and moisture exchangers
ICU: Intensive care units
LCP: Low capacity pump
MMAD: Mass median aerodynamic diameter
ROI: Regions of interest
RT: Respiratory tract
SD: Standard deviation
SPECT/CT: Single photon emission computed tomography/computed tomography
TISAB: Total ionic strength adjustment buffers
