# Supplementary material for: Impact of gas humidification and nebulizer position under invasive ventilation: preclinical comparative study of regional aerosol deposition
Source: Sci Rep. 2023 Jul 8;13:11056. doi: 10.1038/s41598-023-38281-9 (PMC10329710; doi:10.1038/s41598-023-38281-9)
Supplement: Supplementary file 1 — Supplementary Information. [file 41598_2023_38281_MOESM1_ESM.docx]

**Online Data Supplement**

**Title:** Impact of gas humidification and nebulizer position under invasive ventilation: comparative study of regional aerosol deposition.

**Authors:** Yoann Montigaud^1^, Quentin Georges^2^, Lara Leclerc^1^, Anthony Clotagatide^3^, Aurore Louf-Durier^2^, Jérémie Pourchez^1^, Nathalie Prévôt^3, 4^, Sophie Périnel Ragey^2, 4*^.

**Affiliations:**

^1^ Mines Saint-Etienne, Univ Jean Monnet, INSERM, U 1059 Sainbiose; Centre CIS, F - 42023 Saint-Etienne, France.

^2^ Intensive care unit G; CHU Saint-Etienne, Saint-Etienne, F-42055, France.

^3^ Nuclear medicine unit; CHU Saint-Etienne, Saint-Etienne, F-42055, France.

^4^ Université Jean Monnet, Mines Saint-Etienne, INSERM, U1059 Sainbiose, Saint-Etienne, F-42023, France.

*Corresponding author. Email: sophie.perinel.ragey@univ-st-etienne.fr

**RESULTS: supplementary data**

**Deposited fractions**

**Fig S1: Overall comparison of each deposited fraction of nebulized dose in the different parts of the setting for the five conditions.** ETT: endotracheal tube; RT: respiratory tract; HH: heated humidifer.

Conditions D represent non humidifed conditions, whereas conditions H refers to humidified conditions.Subsequent differenciations of conditions are defined according to the nebulizer position as described in *Figure 3*.


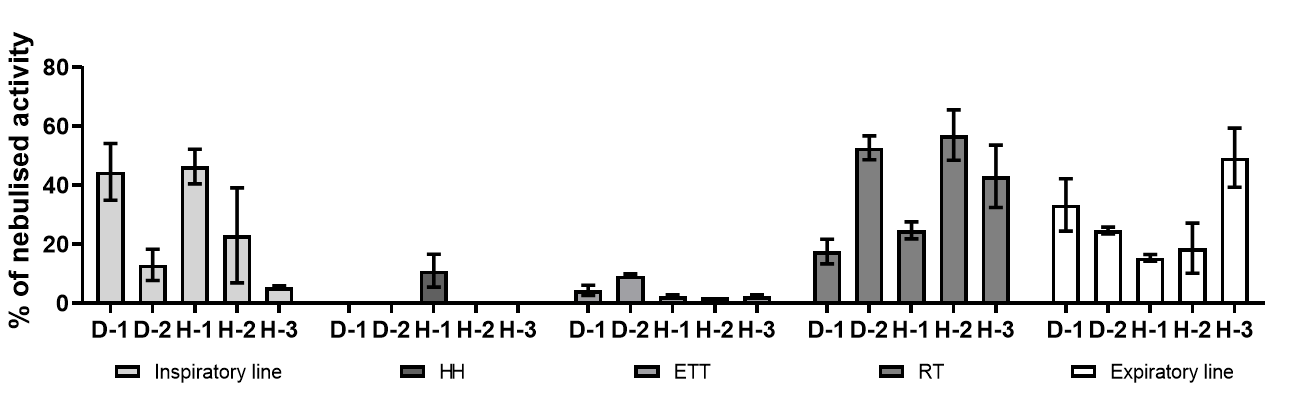


**Table S1: Tukey’s multiple comparison test of respiratory tract deposited fractions according to the different conditions.** Conditions D represent dry and cold conditions, whereas conditions H refers to heated and humidified conditions. Subsequent differenciations of conditions are defined according to the nebulizer position as described in *Figure 3*. In bold lettering the statistical significant differences. P < 0.05 was considered as significant.

| Condition | Adjusted p value |
| --- | --- |
| **D1 vs. D2** | **<0.0001** |
| D1 vs. H1 | 0.4689 |
| **D1 vs. H2** | **<0.0001** |
| **D1 vs. H3** | **<0.0001** |
| **D2 vs. H1** | **<0.0001** |
| D2 vs. H2 | 0.9081 |
| D2 vs. H3 | 0.3108 |
| **H1 vs. H2** | **<0.0001** |
| **H1 vs. H3** | **0.0044** |
| H2 vs. H3 | 0.0508 |

**Respiratory tract distribution**

**Table S2: Tukey’s multiple comparison test central and peripheral distribution of respiratory tract deposited fractions according to the different conditions.**

Conditions D represent non humidifed conditions, whereas conditions H refers to humidified conditions. Subsequent differenciations of conditions are defined according to the nebulizer position as described in *Figure 1*. In bold lettering the statistical significant differences. P < 0.05 was considered as significant.

| Condition | Adjusted p value | |
| --- | --- | --- |
| Central distribution | | |
| **D1 vs. D2** | | **<0.0001** |
| **D1 vs. H1** | | **<0.0001** |
| **D1 vs. H2** | | **<0.0001** |
| **D1 vs. H3** | | **<0.0001** |
| D2 vs. H1 | | >0.9999 |
| D2 vs. H2 | | 0.2349 |
| D2 vs. H3 | | 0.7675 |
| H1 vs. H2 | | 0.2776 |
| H1 vs. H3 | | 0.8205 |
| H2 vs. H3 | | 0.8673 |
| Peripheral distribution | | |
| **D1 vs. D2** | | **<0.0001** |
| **D1 vs. H1** | | **<0.0001** |
| **D1 vs. H2** | | **<0.0001** |
| **D1 vs. H3** | | **<0.0001** |
| D2 vs. H1 | | >0.9999 |
| D2 vs. H2 | | 0.2349 |
| D2 vs. H3 | | 0.7675 |
| H1 vs. H2 | | 0.2776 |
| H1 vs. H3 | | 0.8205 |
| H2 vs. H3 | | 0.8673 |

**Size distribution**

**Table S3: Tukey’s multiple comparison test of MMAD according to the different conditions.**

Neb: nebulizer alone; Conditions D represent non humidifed conditions, whereas conditions H refers to humidified conditions. Subsequent differenciations of conditions are defined according to the nebulizer position as described in *Figure 1*. In bold lettering the statistical significant differences. P < 0.05 was considered as significant. MMAD: mass median aerodynamic diameter.

| Tukey's multiple comparisons test | Adjusted p Value |
| --- | --- |
| Neb vs. D1 | 0.7672 |
| **Neb vs. H1** | **<0.0001** |
| **Neb vs. H2** | **<0.0001** |
| Neb vs. D2 | >0.9999 |
| **Neb vs. H3** | **<0.0001** |
| **D1 vs. H1** | **<0.0001** |
| **D1 vs. H2** | **<0.0001** |
| D1 vs. D2 | 0.8036 |
| **D1 vs. H3** | **<0.0001** |
| **H1 vs. H2** | **0.0335** |
| **H1 vs. D2** | **<0.0001** |
| **H1 vs. H3** | **0.0086** |
| **H2 vs. D2** | **<0.0001** |
| H2 vs. H3 | 0.961 |
| **H3 vs. D2** | **<0.0001** |
